# Comment on Ultrasound Guidance for Botulinum Neurotoxin Chemodenervation Procedures. *Toxins* 2017, *10*, 18—Quintessential Use of Ultrasound Guidance for Botulinum Toxin Injections—Muscle Innervation Zone Targeting Revisited

**DOI:** 10.3390/toxins10100396

**Published:** 2018-09-28

**Authors:** Bayram Kaymak, Fevziye Ünsal Malas, Murat Kara, Arzu Yağız On, Levent Özçakar

**Affiliations:** 1 Department of Physical and Rehabilitation Medicine, Hacettepe University Medical School, Ankara 06100, Turkey; bayramkaymak@yahoo.com (B.K.); mkaraftr@yahoo.com (M.K.); lozcakar@yahoo.com (L.Ö.); 2 Ankara Physical Medicine and Rehabilitation Training and Research Center, Ankara 06100, Turkey; 3 Department of Physical and Rehabilitation Medicine, Ege University Medical School, İzmir 35100, Turkey; arzuon@gmail.com

**Keywords:** botulinum toxin, innervation zone, ultrasound

## Abstract

Recently, the importance of targeting structures during botulinum neurotoxin applications has been discussed in a variety of disorders, including spasticity and dystonia. In this respect, the advantages of ultrasound imaging to traditional techniques have been emphasized. We would like underscore the importance of ultrasound guidance, with targeting innervation zone(s) of the over-active muscles to achieve effective clinical outcomes. Additionally, we also clarify the difference between the terms—innervation zone (motor end plate) and motor point—which have been used by the authors as if they were the same. Further, we disagree with the authors about the intramuscular botulinum neurotoxin application techniques i.e., in-plane vs. out-of-plane whereby the former is, for sure, superior.

We have taken an interest in the review paper by Alter and Karp [1], which has been recently published in your journal. While we congratulate the authors for their great effort in drawing attention to the expanding use of ultrasound (US) imaging for various botulinum toxin (BoNT) applications, we wish to put emphasis on particular issues.

First and foremost, although it is well established that US-guided BoNT injections are performed with better accuracy, there is still discussion on which part of the muscle should be the most appropriate target. In this regard, important US guides based on the innervation zone targeted by BoNT injections have been published [2,3,4,5]. Thereby, more effective (better targeting with lower doses) injections have been proposed. Herein, we need to emphasize that the mentioned innervation zones (motor end plate) harboring the neuromuscular junctions are not the motor points that are referred in the paper by Alter and Karp. Instead, the motor point is physiologically described as the area where the muscle contraction can be created via stimulation with a minimal intensity and short duration of electrical stimulation; and anatomically described as the point(s) where the motor nerve enters a muscle [2]. Herewith, these motor points can be appropriate for nerve blocks (e.g., with phenol) rather than chemodenervation with BoNT. Of note, if one aims to perform nerve blocks, selective motor nerve imaging can also be possible with the use of US [6]. This is actually something denied by the authors.

Second, concerning particular muscles, we strongly disagree with the authors regarding tibialis posterior, iliopsoas, and sternocleidomastoid injections. In the former two muscles, the innervation zones are well known to be localized much more proximal [4,7,8], necessitating injections accordingly (Figure 1 and Figure 2). For the latter one, again based on the evidence of innervation zone distribution, latero-medial rather than cranio-caudal injection would be crucial (Video 1). Yet, the innervation zones are shown to be arranged perpendicular to the muscle fibers [5].

Third, the authors mention that the out-of-plane technique is sometimes more practical and can be preferred for different reasons [1]. However, to follow the precise localization of the needle (tip) either during insertion or while seeding the BoNT toxin through the innervation zones, one needs to continuously follow the aforementioned details of the procedure [9]. This is also paramount for avoiding injury to nearby vital structures.

Last, but not least, the authors refer to a previously published guide and state that high volume injections would be effective in the proximal upper extremity muscles [1]. Herein, we need to mention the contrary i.e., using lower volumes/doses could definitely be more reasonable (cost-effective and with less side effects) as long as the injections target the innervation zone(s) of each/every muscle.

## Figures and Tables

**Figure 1:**
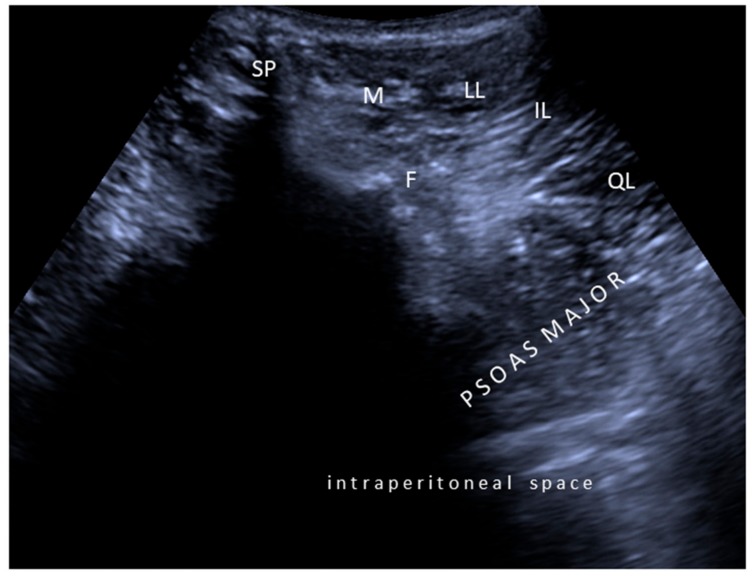
Ultrasound imaging (axial view) for psoas major muscle. SP: Spinous process; F: Facet joint; M: Multifidus; LL: Longissimus lumborum; IL: Iliocostalis lumborum; QL: Quadratus lumborum.

**Figure 2:**
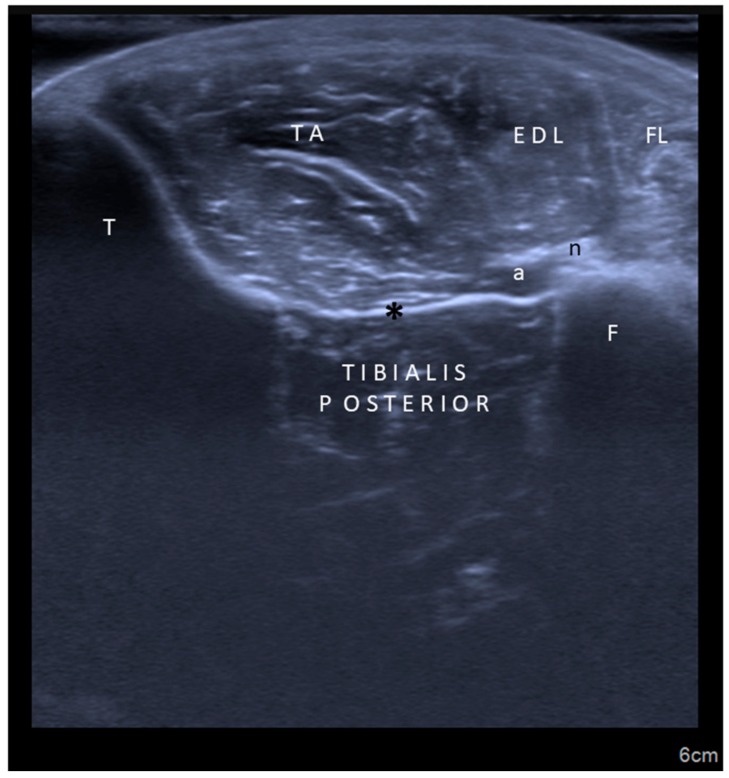
Ultrasound imaging (axial view) for tibialis posterior muscle. TA: Tibialis anterior; T: Tibia; F: Fibula; EDL: Extensor digitorum longus; FL: Fibularis longus; a: Artery; n: Nerve; *: Interosseous membrane.

## Supplementary Materials

The following are available online at http://www.mdpi.com/2072-6651/10/10/396/s1, Video S1: Scm injection.
